# Replication and Inhibitors of Enteroviruses and Parechoviruses

**DOI:** 10.3390/v7082832

**Published:** 2015-08-10

**Authors:** Lonneke van der Linden, Katja C. Wolthers, Frank J.M. van Kuppeveld

**Affiliations:** 1 Laboratory of Clinical Virology, Department of Medical Microbiology, Academic Medical Center, University of Amsterdam, Meibergdreef 15, Amsterdam 1105 AZ, The Netherlands; E-Mail: k.c.wolthers@amc.uva.nl; 2 Virology Division, Department of Infectious Diseases and Immunology, Faculty of Veterinary Medicine, Utrecht University, Yalelaan 1, Utrecht 3584 CL, The Netherlands; E-Mail: f.j.m.vankuppeveld@uu.nl

**Keywords:** enterovirus, human parechovirus, replication, antiviral, small molecules, inhibitor

## Abstract

The *Enterovirus* (EV) and *Parechovirus* genera of the picornavirus family include many important human pathogens, including poliovirus, rhinovirus, EV-A71, EV-D68, and human parechoviruses (HPeV). They cause a wide variety of diseases, ranging from a simple common cold to life-threatening diseases such as encephalitis and myocarditis. At the moment, no antiviral therapy is available against these viruses and it is not feasible to develop vaccines against all EVs and HPeVs due to the great number of serotypes. Therefore, a lot of effort is being invested in the development of antiviral drugs. Both viral proteins and host proteins essential for virus replication can be used as targets for virus inhibitors. As such, a good understanding of the complex process of virus replication is pivotal in the design of antiviral strategies goes hand in hand with a good understanding of the complex process of virus replication. In this review, we will give an overview of the current state of knowledge of EV and HPeV replication and how this can be inhibited by small-molecule inhibitors.

## 1. Enterovirus and Parechovirus Associated Diseases

### 1.1. Picornaviridae

**Figure 1:**
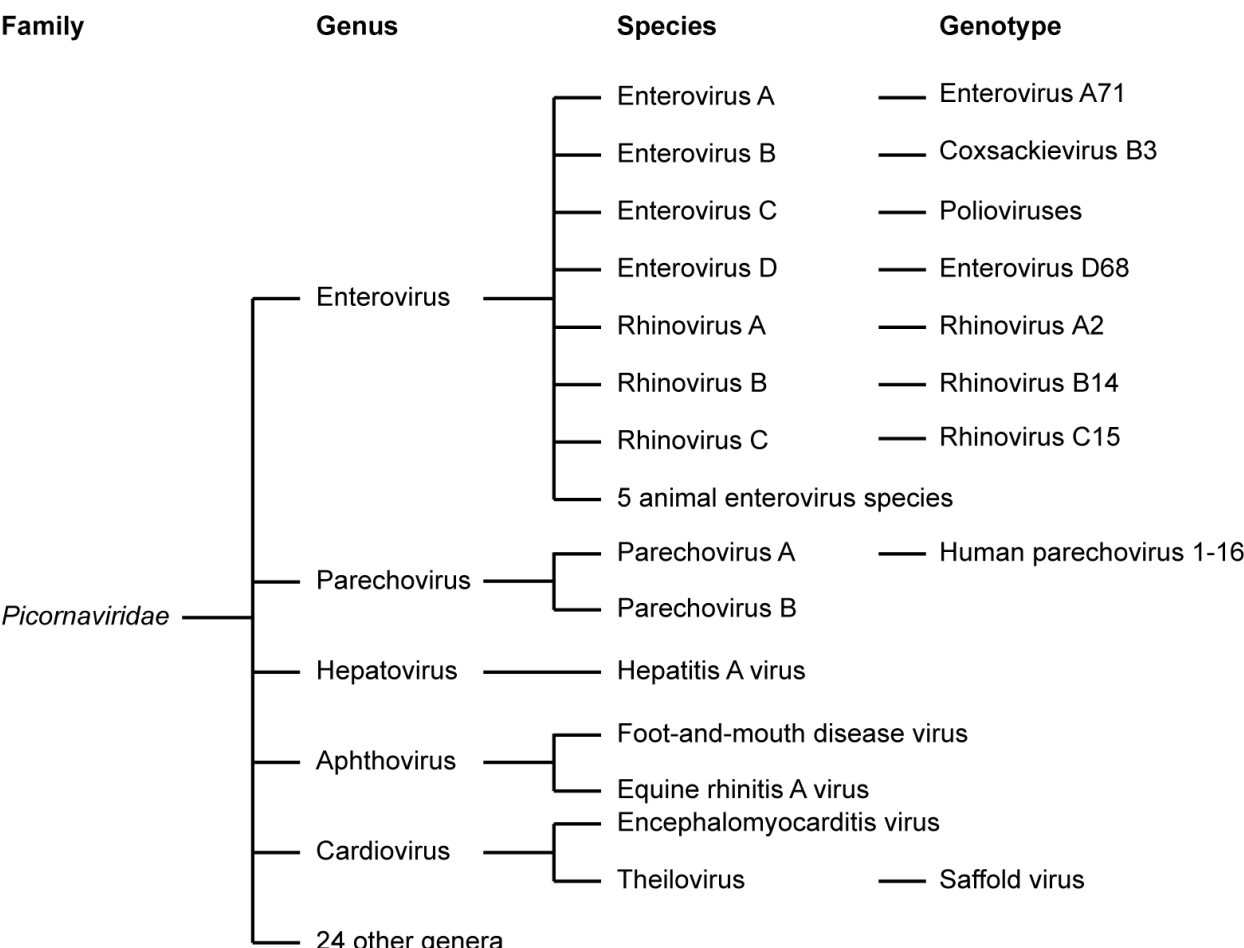
Classification of the virus family *Picornaviridae*. The clinically most important genera are depicted. For a selection of these, the species and some examples of genotypes/serotypes are given.

The virus family *Picornaviridae* is one of the largest virus families (Figure 1), classified into 29 genera (Figure 1) [1,2]. This review focuses on human pathogens belonging to the genera *Enterovirus* and *Parechovirus*. We will discuss the replication strategies of these viruses in the light of antiviral therapy.

### 1.2. Enteroviruses

The genus *Enterovirus* (EV) of the picornavirus family contains many important human pathogens, which are among the most common infections in mankind. Overall, it was estimated that around 10–15 million symptomatic EV infections occur annually in the United States alone [3]. This figure excludes the very prevalent rhinovirus infections. The EVs have been classified into 12 species, including EVs A-D, RV A-C, and five EV species that only infect animals (EV-E to EV-J) (Figure 1) [1,2]. These viruses include coxsackie A and B viruses (CVA and CVB, respectively), echoviruses, polioviruses (PVs), numbered EVs, and rhinoviruses (RV).

EVs are transmitted via the fecal-oral route or via respiratory transmission, depending on the type. EVs have two primary replication sites, the gastrointestinal tract and the respiratory tract, from where the virus can spread to the target organs via the blood circulation. This can result in severe, potentially fatal diseases.

PV is the prototype EV. As in many EV infections, PV infections are mostly clinically mild [4]. However, PV infections can progress to non-paralytic aseptic meningitis (in 1%–2% of cases) or to poliomyelitis, a form of flaccid paralysis (in 0.1%–1% of cases) [4]. Due to intense vaccination programs and surveillance, PV has nearly become extinct, but nevertheless, the virus remains endemic in three countries (Afghanistan, Nigeria and Pakistan) and sporadic PV outbreaks occur.

Coxsackie A and B viruses, echoviruses, and the numbered EVs are associated with a great variety of manifestations, varying from mild respiratory and gastrointestinal infections, herpangina, and hand-foot-and-mouth disease (HFMD), to more severe disease like pleurodynia, hepatitis, myopericarditis, pancreatitis, meningitis, encephalitis, paralysis, and neonatal sepsis leading to mortality [5]. EVs are the most important cause for viral meningitis, accounting for 85%–95% of all cases for which an etiological agent was identified [6].

The genotype/serotype EV-A71 is an emerging pathogen that has caused several outbreaks since the late 1990s. EV-A71 epidemics have been reported worldwide, but mostly in the Asia-Pacific region [7]. HFMD is the most common manifestation of EV-A71 and affects mostly children and infants. However, EV-A71 infections may also result in severe diseases such as pulmonary edema and neurological complications, which may be fatal.

EV-D68 has recently drawn attention because of an outbreak in the United States and to a smaller extent in the rest of the world (e.g., [8,9,10,11]. These EV-D68 infected patients presented with severe respiratory illness. Furthermore, the virus was frequently detected in patients with AFP, suggesting the virus may in rare cases be neurotropic [8,12].

RVs can infect both the upper and the lower respiratory tract and are the major cause of the common cold. Though on the less severe side of the spectrum of the diseases caused by EVs, the common cold results in major costs by, among other things, loss of working days, amounting in the United States to $40 billion annually [13]. In addition to the common cold, RVs can cause severe lower respiratory tract infections, such as pneumonia and bronchiolitis [5]. Moreover, RV infections are a serious threat to patients with asthma, chronic obstructive pulmonary disorder (COPD), or cystic fibrosis in whom respiratory tract infections with RVs can lead to exacerbations [14,15,16,17,18,19,20,21,22,23,24]. RVs are subdivided into the species A, B and C. RV-C has been discovered only recently with the help of molecular diagnostic techniques. Initial studies suggested that RV-C is associated with more severe lower respiratory disease than the other species, but later reports suggest that RV-A may be equally pathogenic [25].

### 1.3. Parechoviruses

When the HPeVs were first identified they were initially classified in the *Enterovirus* genus as echovirus 22 and 23 on the basis of cell-culture characteristics. However, phylogenetic analysis showed these viruses to be genetically distinct from any other picornavirus genus [26,27] and these strains were reclassified in a new genus named *Parechovirus* [28]. Currently, the species Parechovirus *A* contains 16 HPeV types [1,2]. HPeV prevalence has been underestimated, but current data show that HPeVs are at least as prevalent as EVs [29] and that HPeV is a major pathogen in young children [30]. The most commonly circulating HPeV is HPeV-1, which mainly causes mild gastrointestinal and respiratory disease although sometimes in young children more severe disease can be observed [31]. HPeVs are the second most important cause of viral sepsis-like illness and meningitis in infants [32,33,34]. The majority of these cases are caused by HPeV-3 [33], which is the most pathogenic HPeV type. It is associated with paralysis, neonatal sepsis-like illness and sudden death in infected infants [33,35,36,37,38,39,40,41,42,43]. The HPeVs have received very little attention from the scientific community in the past, but continuing reports of HPeV circulation all over the world are increasing awareness of the significance of this virus group.

### 1.4. The Need for Antiviral Drugs against Enteroviruses and Parechoviruses

Although EVs and HPeVs represent a major medical threat, the tools available to fight these viruses are limited. Vaccines are only available against PV: An inactivated PV vaccine (IPV) and an attenuated oral PV vaccine (OPV). Worldwide vaccination schemes started in 1988 have managed to reduce the incidence of PV enormously, but not completely [44]. Sporadic outbreaks may be caused by wildtype virus strains or by vaccine-derived poliovirus (VDPV), which originates from the attenuated OPV. Therefore, as long as OPV is used, the risk of epidemics with VDPV remains. In addition, significant advances have been made towards the development of a vaccine against EV-A71, leading to three inactivated whole virus vaccines that have completed Phase III clinical trials and have applied for approval from the Chinese Food and Drug Administration [45,46,47]. Although vaccine development for specific pathogens such as PV and EV-A71 is possible, developing vaccines against all members of the EV and Parechovirus genera is not feasible due to the sheer quantity of serotypes (more than 250 EVs, and 16 HPeVs) [2].

To date, no antiviral drugs have been approved for the treatment of picornavirus infections, so treatment is currently limited to supportive care. The only option available for treatment is the administration of pooled immunoglobulin from multiple blood donors (intravenous immunoglobulin, IVIG), but success of this treatment modality depends on the presence of specific neutralizing antibodies in the preparation [48,49,50,51]. Hence, there is a strong need for broad-range antiviral drugs to combat EV and HPeV infections. In addition to treating infected patients, antiviral drugs might aid in the eradication of PV by helping to contain VDPV outbreaks and by treatment of immunodeficient patients who are chronically shedding PV. In addition, antiviral drugs may be useful to contain posteradication outbreaks of PV [52].

For the development of antiviral drugs, fundamental knowledge is required about the replication cycle of EVs and HPeVs. Several aspects of virus replication will be summarized in the next section, where the focus will be on the replication of EVs since these viruses have been studied much more intensively and in more detail.

## 2. Enterovirus Replication Cycle

### 2.1. Enterovirus Virions and Viral Genome Organization

Picornaviruses are small positive-strand RNA viruses. The genome is encapsidated by an icosahedral capsid, forming a virion of around 30 nm in size without an envelope.

**Figure 2:**
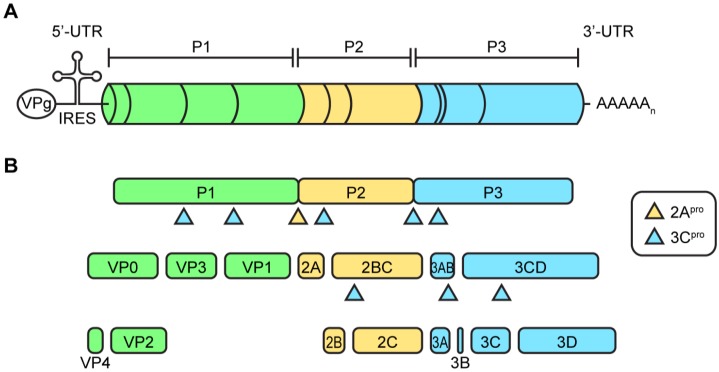
Enterovirus genome. (**A**) Depicted is a schematic representation of the enterovirus genome on scale. The enterovirus genome encodes a single polyprotein divided into a P1, P2, and P3 area. At the 5′- and 3′-end the genome contains untranslated regions (UTR), which are highly structured. The 5′-UTR contains an internal ribosomal entry site for cap-independent translation. At the 5′-end, the RNA genome is covalently bound to the viral protein VPg which is used as a primer during RNA replication; (**B**) The polyprotein is processed into the viral proteins and some stable precursors by the viral proteases 2A^pro^ and 3C^pro^ (and its precursors).

The viral genome contains a single open reading frame with highly structured untranslated regions (UTR) at the 5′- and 3′-end and a 3′-poly(A) tail (Figure 2A). The viral genome is uncapped and instead the 5′-end is covalently coupled to the viral protein 3B, in this context usually termed VPg (viral protein genome-linked). The 5′-UTR contains an internal ribosomal entry site (IRES) which mediates cap-independent translation. Overall, the organization of the open reading frame is similar in all picornaviruses, but there are some differences between genera. In the case of EVs, the open reading frame encodes a polyprotein that contains structural proteins (VP1-4) in the P1 region and the nonstructural proteins (2A–2C and 3A–3D) in the P2 and P3 regions (Figure 2B).

### 2.2. Protein Translation and Processing

**Figure 3:**
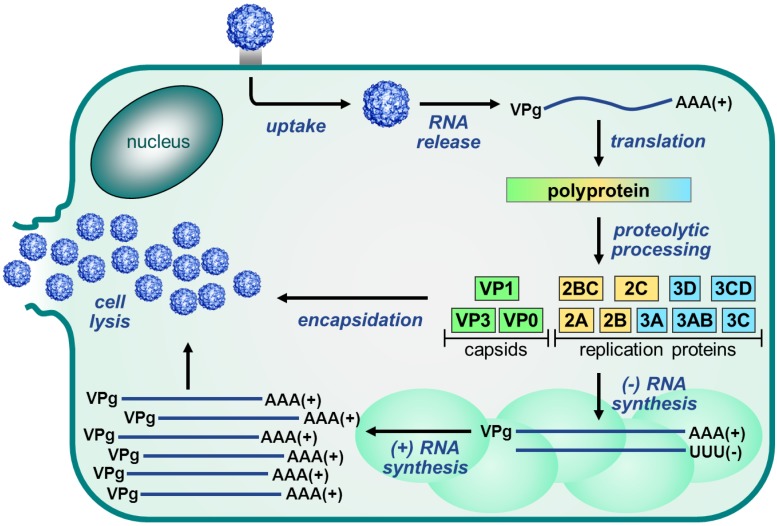
Enterovirus replication cycle. The Enterovirus replication cycle is initiated by binding of the virus to the receptor and internalization into the cell. Subsequently, the viral RNA genome is released from the virion and translated into a single polyprotein which is then processed by the viral proteases to release the viral proteins. Next, the nonstructural proteins mediate the replication of the RNA genome via a negative-stranded intermediate. This takes place on replication organelles that are formed as a result of a rearrangement of cellular membranes. Newly synthesized positive-stranded RNA molecules can then either enter another round of translation and replication (not depicted) or they can be packaged into the viral capsid proteins to form new infectious virus particles which are released upon cell lysis and through several non-lytic mechanisms.

The EV replication cycle, depicted in Figure 3, is initiated by binding to a receptor. The receptor used differs per virus [53]. For many EVs, the receptor binds at a depression in the capsid called the canyon, which surrounds the fivefold axis of symmetry. Subsequently, the virus is internalized and the viral RNA is released into the cytoplasm. The single polyprotein that is produced, is proteolytically processed by the viral proteases 2A^pro^ and 3C^pro^ to release the structural and nonstructural viral proteins and some stable precursors (Figure 2B).

Apart from processing of the viral protein, the viral proteases cleave cellular targets, which serves to optimize the environment for viral proliferation. Cleavage of eIF4G and poly(A)-binding protein (PABP) by 2A^pro^ and 3C^pro^ results in a blockage of translation of cellular proteins, a so-called host shut-off [54,55,56]. In addition, viral proteases cleave several other cellular factors in order to support virus reproduction and/or suppress innate antiviral responses [57,58,59,60,61,62].

### 2.3. Enteroviral RNA Replication

Once liberated from the polyprotein, the nonstructural proteins mediate the replication of the viral genome. RNA genome replication is initiated by uridylylation of the protein primer VPg by the viral RNA-dependent RNA polymerase 3D^pol^ using a secondary RNA structure in the viral genome called cis-acting replication element (Cre) as a template [63,64,65,66]. VPgpUpU is then elongated by 3D^pol^ to produce a negative-stranded intermediate which in turn is used as a template for synthesis of positive-stranded RNA molecules. Positive-stranded RNA molecules can then either enter another round of translation and replication or they can be packaged into capsids to produce infectious virus particles.

### 2.4. The Role of Viral Proteins and Host Factors in Membrane Rearrangements

**Figure 4:**
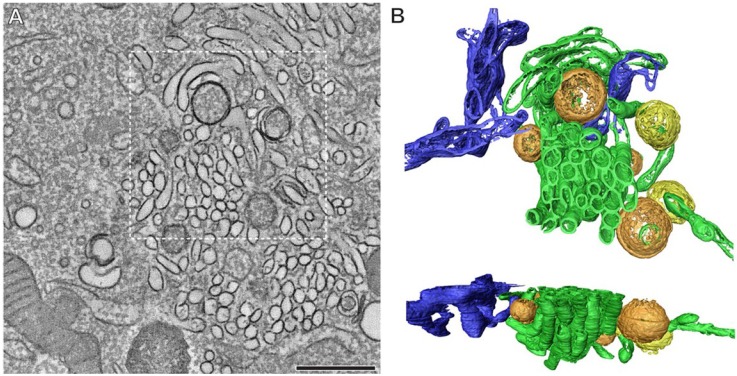
Extensive membrane rearrangements in Enterovirus-infected cells. An electron tomographic slice through a serial tomogram, bar = 500 nm (**A**); and top and side views of the surface-rendered model of the boxed area (**B**) show the presence of single-membrane tubules (**green**), open (**orange**) and closed (**yellow**) double-membrane vesicles in a cell infected with coxsackievirus B3 at 5 h post infection. The ER is depicted in blue. Reprinted from Limpens *et al.* [70], mBio 2011 with permission from the authors, © 2011 by the American Society for Microbiology.

Typical for positive-strand RNA viruses, replication of the viral RNA takes place on cellular membranes which are drastically reorganized during virus infection [67,68]. In EV-infected cells, both single- and double-membrane structures are observed (Figure 4) [69,70]. Electron tomography studies with PV and CVB3 have revealed that early in virus infection (when RNA replication is already maximal) single-membrane tubular structures are predominant, whereas in later stages these structures appear to flatten, curve, and fuse to form double-membrane vesicles (DMV) [69,70]. These DMVs can then be wrapped by multiple additional cisternae and form multilamellar structures.

The exact origin of the membranes of these organelles is yet unclear. Evidence has been presented that the membranes originated from the early secretory pathway while other data suggested they were derived from the autophagy pathway. The results from the electron tomography studies suggest that there may be some truth in both theories, with the early secretory pathway acting as a source for the single membrane tubules and the autophagy pathway being involved in DMV formation.

**Figure 5:**
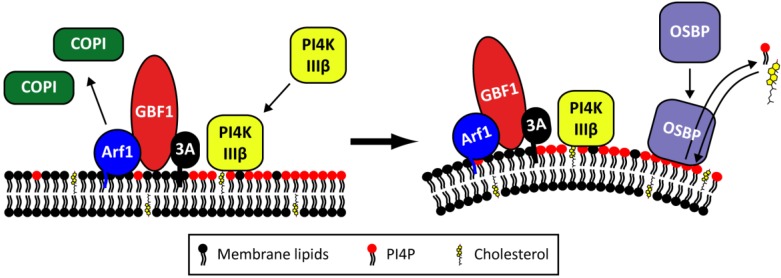
The proposed role of Golgi-localized host factors in Enterovirus replication. Upon infection, the viral 3A protein recruits GBF1 and indirectly Arf1 to the replication organelles. As a result, COP-I is lost from the membranes. At the same time, PI4KIIIβ is recruited by 3A through a GBF1/Arf-independent mechanism, resulting in an increase in PI4P lipids. OSBP then binds to the PI4P lipids and mediates a PI4P/cholesterol counterflow between the membranes of the replication organelles and the ER.

Important observations that support a role for the early secretory pathway in the membrane rearrangements are that Brefeldin A (BFA), a well-known inhibitor of ER-to-Golgi transport, completely abolishes EV replication [71,72,73,74] and that several proteins from the secretory pathway are essential for virus replication and can be detected on replication organelles. One of these is Golgi-specific BFA-resistance factor 1 (GBF1), which is a target of BFA. In uninfected cells, GBF1 stimulates GTP exchange of the GTPase ADP-ribosylation factor 1 (Arf1), which is localized on the Golgi complex and the ER-Golgi intermediate compartment. Upon activation, Arf1-GTP becomes membrane-bound and mediates the recruitment of effector proteins such as the COP-I coat complex, thereby inducing the formation of secretory vesicles. Arf1 is thus a key regulator of protein and lipid transport within the early secretory pathway. Upon infection, the viral protein 3A recruits GBF1 and indirectly Arf1 to replication organelles (*i.e.*, virus-induced vesicles plus associated replication complexes) through a direct interaction with GBF1 (Figure 5) [71,75,76]. Through a yet unknown mechanism, this leads to the loss of COP-I from membranes, resulting in a disturbance of the secretory pathway and blockage of protein secretion [75,77,78,79]. This impairs the expression of MHC class I on the cell surface and cytokine secretion [80,81], implying that the virus-induced membrane rearrangement not only serves to facilitate viral RNA replication but also to suppress infection-limiting host immune responses.

Phosphatidylinositol-4-kinase III beta (PI4KIIIβ), another Golgi-localized protein, is also an essential host factor for EV replication [77]. PI4KIIIβ is a kinase that synthesizes phosphatidylinositol-4-phosphates (PI4P). As a precursor for PI(4,5)P_2_, PI4P lipids are part of PI3K and phospholipase C signaling pathways, but PI4P lipids also recruit proteins with a PI4P-binding pleckstrin homology (PH) domain to membranes, thereby regulating membrane biogenesis, lipid homeostasis, and vesicle-mediated trafficking at the Golgi complex [82,83,84]. During infection, PI4KIIIβ is recruited to replication sites by 3A and consequently local levels of PI4P lipids increase (Figure 5) [77]. The mechanism behind the recruitment of PI4KIIIβ by 3A remains to be determined but was shown to be independent of GBF1, Arf1, and ACBD3 [85,86]. *In vitro*, PI4P lipids specifically bound 3D^pol^, suggesting that they may serve to recruit the viral polymerase to replication complexes [77], but firm proof for this idea is lacking. Alternatively, or additionally, the function of PI4P lipids in virus replication may be to recruit PH domain-containing proteins for example with membrane-modifying properties.

One such protein appears to be oxysterol-binding protein (OSBP), a PI4P-binding protein that transports PI4P lipids produced by PI4KIIIβ from the Golgi complex to the ER, in exchange for cholesterol which is transported in the opposite direction [87]. Recent studies by others and ourselves have revealed that OSBP binds to PI4P-enriched replication organelle membranes and mediates a PI4P/cholesterol counterflow between these membranes and the ER (Figure 5) [88,89,90]. As a result, the cholesterol content of membranes of the replication organelles is increased. In addition to this mechanism, increased uptake of cholesterol and a role of endosomal cholesterol have been suggested to contribute to the accumulation of cholesterol in the membranes of the replication [90,91,92]. The cholesterol content of membranes determines the membrane fluidity and formation of lipid microdomains and therefore the virus-induced accumulation of cholesterol may serve to induce the membrane deformations required to generate replication organelles. All in all, it appears that the regulation of PI4P and cholesterol levels is very important to support replication.

DMVs are reminiscent of autophagosomes with respect to their appearance and formation, which originated the idea that the autophagic pathway is involved in the formation of replication organelles. The recent observation that DMVs occur mostly in later stages of infection suggests that this pathway is mostly important in the advanced stages of membrane rearrangements [69,70]. Inhibition of the autophagy pathway impairs viral replication, but only to a modest extent [93,94]. A recent publication has provided evidence that vesicular acidification promotes maturation of PV particles (*i.e.*, VP0 cleavage, see next section), implicating a role for autophagy and DMVs in the last step of the replication cycle [95]. Furthermore, it has been suggested that the DMVs might be involved in non-lytic release of virus particles by fusion with the plasma membrane, challenging the dogma that EVs egress only through cell lysis. Together, these data suggest that the early secretory pathway and the autophagy pathway have a distinct, but important function during EV replication.

Genetic and biochemical evidence suggests that the viral proteins 2BC and 3A are involved in the formation of replication organelles during infection [96,97,98]. These proteins have hydrophobic domains and extensively interact with cellular membranes. 3A is probably important in membrane reorganization through its (direct and indirect) interactions with GBF1, Arf1, and PI4KIIIβ. 2B is a viroporin that enhances the permeability of ER and Golgi membranes [99,100,101,102,103]. Overexpression of 2B leads to disturbed ion homeostasis, impaired transport of proteins through the Golgi complex, and increased targeting of endocytic vesicles to the Golgi complex [78,101,104,105]. How and if these activities are involved in the formation of replication organelles is unknown. 2C has been postulated to contribute to the membrane remodeling by insertion of its hydrophobic domains in the membranes, as well as through its interaction with reticulon proteins [106]. These latter proteins are membrane-shaping proteins that induce and stabilize positive membrane curvature, and may be involved in the formation of the positively curved membranes that are essential for the morphogenesis of the viral replication organelles.

As has become clear from this brief overview, membrane remodeling involves many viral and host proteins and lipids and is a very complicated process that is not completely understood.

### 2.5. Morphogenesis and Virus Release

Once synthesized, viral RNA of positive polarity is encapsidated by capsid proteins to form new virions. This process is coupled to active replication as only newly synthesized genomes are encapsidated [107,108]. This is not guided by an RNA encapsidation signal or RNA-protein interactions, but rather by an interaction between 2C, which is located in the replication complex, and the capsid protein VP3 [109].

**Figure 6:**
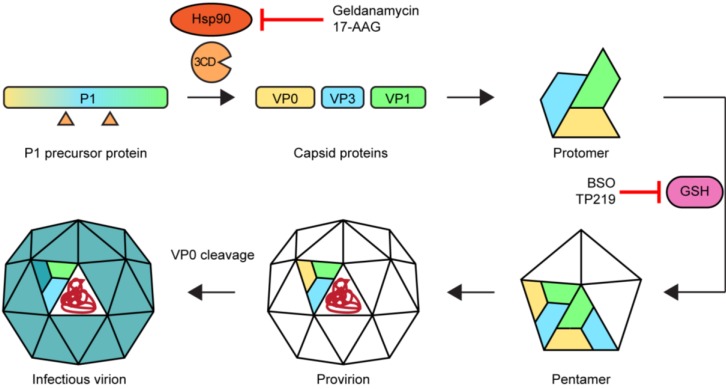
Morphogenesis of enteroviruses and targets for assembly inhibitors. Hsp90 ensures the proper folding of the P1 precursor protein enabling the cleavage by 3CD^pro^ into capsid proteins VP0, VP3, and VP1 which then form protomers. For part of the EVs, glutathione (GSH) is required either for the transition of protomers into pentamers or for the stabilization of pentamers. Twelve pentamers plus the viral genome (in red) combine to form a provirion, followed by a maturation step in which the VP0 protein is cleaved into VP4 and VP2. Treatment with Hsp90 inhibitors or glutathione depletors results in impaired morphogenesis.

The assembly of new virions (Figure 6) is initiated by the release of the P1 capsid precursor from the polyprotein. This is subsequently folded by the chaperone protein Hsp90 and processed by 3CD^pro^ to release VP0 (the precursor of VP4 and VP2), VP1, and VP3 [110,111]. In a spontaneous process, these capsid proteins assemble to form a protomer. Five protomers together then form a pentamer which in turn assemble to form a provirion. Several, but not all, EVs require the presence of glutathione for the formation and/or stability of the pentamers [112,113,114,115]. The last step is a maturation of the virion by RNA-induced cleavage of VP0 into VP2 and VP4, yielding an infectious virus particle. This process has been suggested to be enhanced by the acidic environment in autophagosome-like DMVs [95].

The dogma has always been that newly formed infectious particles are released by lysis of the host cell, but recent reports have suggested additional methods of egress that do not require cell lysis, such as non-lytic release through DMVs and release of phosphatidylserine lipid-enriched vesicles packed with virions [116,117]. This mechanism is reminiscent of the release of hepatitis A virus, another picornavirus, which was recently shown to be released as membrane-wrapped virus particles in membrane structures resembling exosomes [118].

## 3. Antiviral Compounds

Antiviral therapy can have a variety of mechanisms of action and each step of the virus replication cycle can be targeted. Virus replication can be impaired by targeting either viral proteins or host factors that are required for virus replication. Decades of searching for compounds with antipicornaviral activity has yielded a range of compounds that inhibit EV replication, although none of them have reached approval for clinical use. The current status of research on and development of small-molecule antiviral compounds that target the replication of EVs is summarized here below.

### 3.1. Enterovirus Inhibitors

***Capsid binders.*** The three compounds that are currently under clinical evaluation are pleconaril (Viropharma, Exton, USA, licensed to Schering-Plough in 2003), BTA798 (Biota Pharmaceuticals, Alpharetta, USA), and pocapavir (V-073, ViroDefense Inc., Rockville, USA)). All three compounds bind the capsid in the canyon, a depression in the capsid which is responsible for receptor binding. By doing this they impair the first steps in the viral replication cycle, *i.e.*, receptor attachment and/or viral uncoating. Pleconaril displays broad-spectrum activity against most, but not all EVs and had some beneficial effects in clinical trials [119,120,121,122,123,124], but was rejected by the FDA in 2002 for the treatment of the common cold due to safety issues [125]. In a later clinical phase II trial on the prevention of asthma exacerbations and common cold symptoms in asthma patients challenged with RV, pleconaril treatment had no significant effect [126] while the results of a trial to test the efficacy of pleconaril in infants with enteroviral sepsis syndrome have not been made available yet [126,127].

The compound BTA798 is currently under investigation for the treatment of RV infections in asthmatic patients. In March 2012, a clinical phase IIb trial testing the efficacy of BTA798 in asthma patients with naturally acquired RV infections was completed [128]. Biota reported significant reductions in symptoms upon treatment with BTA798 and is currently planning another phase IIb trial in RV-infected patients with moderate-to-severe asthma [129].

Pocapavir is under clinical investigation to be used in the eradication of PV [130], but has also been shown to have activity against other EVs [131]. Recently, a case study was reported in which pocapavir was used to treat an infant with severe enteroviral sepsis [132]. Pocapavir appeared to be tolerated well, but a clear antiviral effect was absent.

One of the biggest issues with capsid binders is that EVs readily develop resistance [133]. The quick emergence of resistance may be one of the reasons that the performance of this class of compounds in clinical trials is disappointing [134]. In addition, one of the problems with capsid binders is that they do not inhibit all EVs [135,136,137] and we have recently reported a naturally occurring pleconaril-resistant E11 strain [50,138]. Because of these resistance issues it is questionable whether capsid inhibitors will make it to the market.

***Protease inhibitors.*** Viral proteases have shown to be useful targets for antiviral therapy in treatments against human immunodeficiency virus (HIV) and hepatitis C virus. The only EV protease inhibitors that have made it to clinical studies are the 3C^pro^ inhibitors rupintrivir (also known as AG7088), and AG7404 (also known as compound 1). These protease inhibitors have been designed to mimic the protease substrate. The compound rupintrivir was developed as a potent inhibitor of RV 3C^pro^, but further studies revealed that rupintrivir also inhibits replication of other EVs [139,140,141,142,143]. Rupintrivir is an irreversible peptidomimetic with an α,β-unsaturated ester. Because of limited activity in clinical trials with natural RV infections [144], the clinical development was halted. AG7404 was developed as an analog of rupintrivir with improved oral bioavailability compared to rupintrivir [145]. AG7404 displays antiviral activity *in vitro* and is safe and well-tolerated *in vivo*, but clinical development was discontinued [144].

***Polymerase inhibitors.*** Evidently, inhibition of the viral polymerase 3D^pol^ is detrimental for virus replication and is therefore an attractive strategy for antiviral approaches. There are two classes of polymerase inhibitors. The nucleoside analogs are compounds that have structural similarity to endogenous nucleosides. Incorporation of these analogs by 3D^pol^ results in chain termination and/or incorporation of incorrect nucleosides leading to mutations. One such compound is ribavirin, a synthetic purin analog. Treatment with ribavirin leads to lethal mutagenesis [146,147]. The other class of polymerase inhibitors encompasses the non-nucleoside inhibitors. These inhibitors can have a variety of mechanisms of action. For example, the polymerase inhibitor amiloride increases the error rate of 3D^pol^ and competes with incoming nucleoside triphosphates and Mg^2+^ [148]. In addition, we have just reported the discovery of a compound (GPC-N114) which interferes with productive binding of the template-primer to 3D^pol^ by binding at the template-binding site [149]. However, for most compounds the mechanism of action has been poorly characterized (gliotoxin, DTrip-22, aurintricarboxilic acid, BPR-3P0128) [150,151,152,153,154]. Up to now, no 3D^pol^ inhibitors have been tested in clinical trials.

***2C-targeting compounds.*** Another group of inhibitors is that of compounds that select for mutations in 2C. The functions of the nonstructural protein 2C are not fully understood, but the protein has been implicated in RNA replication [106,155,156,157,158,159,160], RNA binding [161,162,163], the induction of membrane rearrangements [97,98,103,164], encapsidation [109,165,166], and uncoating [167]. On the basis of several conserved motifs, 2C was also predicted to be a helicase [168], although the RNA unwinding activity has not yet been experimentally confirmed. 2C has a nucleoside triphosphate-binding motif and displays ATPase activity [169,170]. A variety of antiviral compounds have been shown to target 2C (guanidine hydrochloride, fluoxetine, HBB, MRL-1237, and TBZE-029) [171,172,173,174,175,176], but their mechanisms of action have not been resolved yet. This is in a great part due to the inability to obtain functional recombinant 2C and the lack of a crystal structure. None of the 2C inhibitors have progressed to *in vivo* studies in humans.

***PI4KIIIβ inhibitors.*** In the late 1970s, the compound enviroxime was reported to have potent antiviral activity against EVs [177], but it took until 2012 to identify PI4KIIIβ as the target of enviroxime following the recognition of PI4KIIIβ as an essential host factor [77,178]. Aided by cross-resistance of virus variants with mutations in 3A, also GW5074, T-00127-HEV1, and compound 1 were identified as PI4KIIIβ inhibitors [178,179,180,181,182,183]. Enviroxime was tested in several clinical studies in which it was able to reduce symptoms, but overall the *in vivo* efficacy was disappointing [184,185,186,187,188,189]. This in combination with gastrointestinal side-effects and a poor pharmacokinetic profile caused the clinical development to be halted [188].

For a number of other PI4KIIIβ inhibitors little if any toxicity in cell lines as well as in primary human fibroblasts was reported [178,183,190,191]. One of these inhibitors (*i.e.*, compound 1) also showed no obvious adverse effects upon testing in a CVB4-induced pancreatitis mouse model, while it resulted in a strong inhibitory effect on *in vivo* CVB4 replication and CVB4-induced pathology [183]. However, in another study it was described that chemical inhibition of PI4KIIIβ by a small molecule identified by Boehringer Ingelheim as well as by T-00127-HEV1 was lethal to mice [192]. Moreover, PI4KIIIβ inhibitors developed by Novartis were found to interfere with lymphocyte proliferation and chemokine secretion in a mixed leukocyte reaction [190]. These latter results have raised many concerns about the potential application of PI4KIIIβ inhibitors to treat EV infections.

***OSBP inhibitors.*** The 3A-mutant viruses that were resistant against PI4KIIIβ inhibitors, also displayed a resistant phenotype against a set of antiviral compounds that had no effect on PI4KIIIβ activity. This included the compounds itraconazole, OSW-1, AN-12-H5 and T-00127-HEV2, and 25-hydroxycholesterol. We and others revealed that the target of these compounds was OSBP [88,193,194]. Studying the mechanism-of-action of these compounds has been significantly instrumental in discovering that OSBP is required for virus replication. Itraconazole has already been approved by the FDA in 1992 as an antifungal drug and therefore already much is known about the behavior of this compound *in vivo*. In recent years, itraconazole has also gained interest as an anticancer reagent, and is currently being tested in clinical trials [195]. The wealth of information gathered while testing itraconazole for these other applications should facilitate testing the antiviral effect of itraconazole in mouse models.

***Assembly inhibitors.*** Assembly of new virus particles is a stepwise process which can therefore be interrupted at multiple steps (Figure 6). The use of inhibitors has greatly contributed to our knowledge of the assembly process. The chaperone Hsp90 interacts with the P1 capsid precursor and ensures a correct folding that enables recognition and/or cleavage by 3CD^pro^ [110,196]. Inhibition of Hsp90 with the compounds geldanamycin or its analog 17-AAG (17-allyamino-17-demethoxygeldanamycin) still allows the interaction between Hsp90 and P1, but processing of P1 is impaired. As a result, morphogenesis is impaired. The role of glutathione in the assembly of a subgroup of EVs has come to light by the study of the glutathione synthesis inhibitor BSO (buthionine sulfoximine) and the glutathione scavenger TP219 [112,113,114,115]. BSO has been tested in clinical trials for testing its ability to reverse the resistance to chemotherapy in cancer treatments caused by high glutathione levels. These showed that BSO treatments were tolerated relatively well [197,198,199], but to our knowledge, no attempts or plans have been made to test the antiviral activity of BSO or other glutathione-depleting compounds *in vivo*.

### 3.2. Parechovirus Replication and Inhibitors

Despite the clinical significance of HPeVs, these viruses have not been the topic of many in-depth molecular studies and very little work has been done to identify compounds with antiviral activity against this virus group. The fact that HPeVs and EVs are members of the same virus family, suggests that HPeVs may benefit from the ample work being done on EVs. However, work done so far, has only highlighted the differences between the two virus genera.

The capsid binder pleconaril has been described not to possess any antiviral activity against HPeVs [200,201]. Though the HPeV capsid structure is similar to that of EVs in that it is icosahedral and the capsid proteins have the typical β-barrel structure, the HPeV capsid has a shallower canyon than in EVs [202]. Moreover, the canyon does not mediate receptor binding, at least not for the HPeVs that utilize integrins as a receptor [202]. Thus, in this light it is not surprising that pleconaril does not possess antiviral activity against HPeV. Since all of the capsid binders developed against EVs target the canyon, these are not expected to display activity against HPeVs.

Besides capsid inhibitors, 3C^pro^ inhibitors were suggested to be the most promising candidates for anti-HPeV therapy [201]. However, we showed recently that rupintrivir, the best-established EV 3C inhibitor, and SG85, a recently developed analogue [203], do not inhibit the proteolytical activity of HPeV 3C^pro^ [204]. The reason for this is unknown, since the structure of HPeV 3C^pro^ has not been resolved yet. An explanation may lie in the difference between the cleavage sites recognized by the EV and HPeV proteases, since these are peptidomimetics designed to resemble the cleavage site recognized by 3C^pro^. Rupintrivir is a tripeptide-derived molecule with a Michael acceptor moiety [205]. Its structure is based on the leucine-phenylalanine-glutamine peptide sequence at the P3-P2-P1 position observed in EV 3C^pro^ cleavage sites [205,206]. However, none of the HPeV 3C^pro^ cleavage sites observed contain a leucine at P3 [207]. On the other hand, SG85 contains a t-butyl ether of serine at P3, and a serine has been observed at this position in HPeV cleavage sites [203,207]. Though the t-butyl ether modification may be responsible for the inactivity of SG85 against HPeV 3C^pro^, this suggests that differences in cleavage sites alone cannot completely explain the disparity between the susceptibilities of the EV and HPeV 3C proteases to rupintrivir and SG85. Detailed analysis of cleavage sites recognized by HPeV 3C^pro^ and especially crystal structures are required to understand the insusceptibility of HPeV 3C^pro^ to rupintrivir and SG85 and for the design of HPeV 3C^pro^ inhibitors.

In contrast to 2A of EVs, the 2A protein of HPeV does not have proteolytical activity and the P1/P2 cleavage is carried out by 3C^pro^ [208]. Hence, HPeV 2A does not induce a host shut-off through cleavage of translation factors [209]. Instead, 2A has been described to bind the 3′-end of the HPeV genome, suggesting a role in HPeV RNA replication [210]. Accordingly, EV and HPeV 2A have only very limited amino acid identity [211]. HPeV 2A will therefore not benefit from antiviral studies targeting EV 2A^pro^, though these have been rare.

On the other hand, for both genera the 2C protein is predicted to be a helicase and has been shown to have ATPase activity [212]. 2C represents a promising target for EVs, but the EV 2C inhibitors GuHCl and HBB have no effect on HPeV 2C [213]. Since these compounds have the broadest spectrum of anti-enteroviral activity of the 2C inhibitors available [171,175,176,213,214], it is to be expected that the others do not inhibit HPeV 2C either. For fluoxetine and TBZE-029, for example, the susceptibility seems to require the presence of the so-called AGSINA motif, which is not present in HPeV 2C [176].

The replication strategies employed by HPeV are mostly a black box, but it is clear that they are different from those used by EVs. A first clue came from the partial resistance of HPeV to the GBF1 inhibitor BFA, which completely blocks EV replication [72]. In addition, COP-I is dispersed in cells infected with HPeV while in EV-infected cells, COP-I is found on replication organelles [72]. Accordingly, the amino acid identity between HPeV 3A and EV 3A is very low [211] and the replication organelles formed upon infection have a different morphology and appear to have a different origin than those in EV-infected cells [72,215]. It is not known whether HPeV uses PI4KIIIβ like EVs, or any other PI4K for replication. No PI4K inhibitors have been tested with HPeV thus far, but doing so will help to elucidate this question. Our study on itraconazole as an EV and cardiovirus inhibitor, revealed that this compound has no effect on HPeV replication, suggestive of an independence of OSBP or at the least a different usage of OSBP [88].

Despite that multiple compounds have been identified as EV 3D^pol^ inhibitors, none of these have been tested with HPeV. However, it seems reasonable to assume that the nucleoside analogues such as ribavirine will also display activity against HPeV since these have a very broad spectrum of activity as a result of a very conserved nucleotide-binding site [216]. Our preliminary results suggest that GPC-N114 has no effect on HPeV replication (unpublished results).

Other than that the maturation cleavage of VP0 into VP4 and VP2 does not occur in HPeVs, not much is known about assembly of HPeV virions. It has not been studied whether HPeV requires the chaperone Hsp90 and/or glutathione for morphogenesis.

Overall, it is clear that there are large gaps in our knowledge of HPeV biology and that the tools against HPeVs are severely limited.

### 3.3. Issues in Antiviral Drug Development

Several issues complicate the development of antiviral drugs against EVs and HPeVs. First of all, broad-spectrum antiviral activity is desired, to be able to treat all EV or HPeV infections with the same antiviral drug to eliminate the need for typing. In addition, it would be undesirable to have to develop dozens of antiviral drugs to be able to treat all EV/HPeV infections. Secondly, the possible emergence of drug resistance could become a major problem when drugs are introduced into the clinic. The mutation frequency for RNA polymerases is relatively high. As a result, a virus population can be described as a quasispecies. Drug-treatment will select for virus mutants with a selective advantage, allowing the outgrowth of resistant virus mutants and rendering the antiviral therapy ineffective. Although this is considered less of a problem when treating acute infections, the class of capsid binders has shown a quick emergence of resistant variants, making this class of inhibitors less suitable for treating picornaviral infections [133]. Therefore, compounds to which the virus cannot (easily) develop drug resistance are highly needed.

One strategy believed to help with both of the above-named issues, is to develop antiviral drugs that target host factors. Many essential host factors are common to all viruses within a genus, which allows targeting an entire genus without the problem of variable drug pockets in the viral proteins. Part of the host factors may even be shared by multiple genera. One illustrative example of this is the OSBP inhibitor itraconazole, for which the spectrum of antiviral activity encompasses all tested viruses of both the EV and even the cardiovirus genus [88]. In addition, it is highly unlikely that a host factor will become resistant to the drug in response to therapy. A finding that supports this hypothesis, is the inability to obtain resistant PV when passaging virus in the presence of geldanamycin, an inhibitor of the host chaperone protein Hsp90 which is required for virus assembly [110]. However, there are also examples where the virus has become resistant to host protein-targeting compounds. For example, PV could become resistant to the GBF1-inhibitor BFA by acquiring mutations in 2C and 3A [217]. Furthermore, we found that single point mutations in CVB3 3A can render CVB3 independent of PI4KIIIβ/PI4P and resistant to OSBP inhibitors, which shows that RNA viruses can even become resistant to inhibitors of essential host factors and use a bypass mechanism for replication independent of (high levels of) a critical lipid [178].

A potential downside of targeting host factors is that the chances of adverse effects of drug treatment are higher when inhibiting cellular targets than when targeting viral proteins. However, one should realize that in general most drugs have host targets. In fact, this is the case in all diseases in which no pathogen is involved, demonstrating that targeting host proteins is not necessarily problematic. For example, inhibitors of the hepatitis C virus host factor cyclophin A, a protein that makes up 0.1%–0.4% of the total cellular protein content [218], generally have a good safety profile and any adverse effects observed seem to be associated with the specific inhibitor rather than the target [219].

Overall, targeting host factors may prove a good, though not perfect, strategy, notwithstanding the usefulness of direct-acting antiviral compounds. Ultimately, the aim would be to develop combination therapy consisting of multiple antiviral drugs with different resistance profiles.

## 4. Future Perspectives in Antiviral Research

In the last decades, many researchers have focused on identifying inhibitors of EV replication, leading to the discovery of numerous of EV inhibitors. However, since none of these have reached the market, a lot of work still needs to be done.

A category of inhibitors that remains largely unexplored is that of the allosteric 3C^pro^ inhibitors. Most of the protease inhibitors developed so far—such as rupintrivir, compound 1, and the SG compounds—are peptidomimetic active site inhibitors which have been designed based on the cleavage site recognized by the targeted protease. Interest has increased in allosteric site inhibitors, since the occurrence of resistance mutations in the active site of competitive compounds often gives cross-resistance to other competitive inhibitors [220,221]. Last year, Wang *et al.* [222,223] reported the first inhibitors of EV71 3C^pro^ with a binding site outside the substrate binding site, illustrating the potential of small molecules as allosteric inhibitors of EV proteases. Further endeavors are expected to yield more allosteric inhibitors, thereby opening up a whole new class of 3C^pro^ inhibitors.

Another area where advances can be made, are the 2C inhibitors. The fact that many compounds have been found to target 2C implies that 2C is easily targeted, possibly as a result of an easily accessible compound-binding pocket. Elucidation of the structure and function of 2C has proven very difficult, but will greatly benefit the development and characterization of 2C inhibitors. For now, the most promising 2C inhibitor appears to be fluoxetine. Its modest potency in combination with the relatively low plasma concentrations achieved *in vivo* and reported side effects could possibly obstruct the use for antiviral therapy *in vivo* [176]. However, it was recently pointed out that fluoxetine reaches considerably higher concentrations in the brain than in plasma, opening up the possibility to use fluoxetine for treatment of neurological diseases caused by EV-B and EV-D viruses, such as EV-D68 [224].

While considerable progress has been made for the EVs over the past decades, research into HPeV replication and anti-parechoviral therapy is still in early stages. Clearly, anti-parechoviral studies would be greatly facilitated by a greater knowledge of HPeV replication. For example, HPeV 2A may be used as a target for antiviral therapy, once its role in the RNA replication has been deciphered. Currently we are trying to gain more insight into the replication strategies employed by HPeVs by utilizing inhibitors that target EV host factors. This provides an easy and quick method to determine whether these viruses require PI4KIIIβ, OSBP, Hsp90, and glutathione for efficient replication. The results will provide a starting point from where more in-depth replication studies can be done and may provide targets for antiviral therapy. In addition, a crystal structure of HPeV 3C^pro^ would be of great benefit for the design of inhibitors of this enzyme.

For both groups of picornaviruses, the repurposing of drugs, *i.e.*, the use of drugs already on the market for a different indication, may be a way to obtain antiviral drugs that can quickly be put to practice, because these drugs have been well-characterized and tested *in vivo*. Both the antifungal itraconazole and the antidepressant fluoxetine were found when screening FDA-approved drug libraries [88,176], and additional screening will probably yield more hits.

In the coming years, advances made in the fields of EV/HPeV replication mechanisms and antiviral therapy will develop synergistically, yielding a better understanding of replication mechanisms, thus providing novel targets for antiviral therapy and the characterization of compounds with antiviral activity, giving us more insight into aspects of virus replication. Though the development of antiviral therapy represents a major challenge, progress made in recent years raises hope that this is indeed an achievable goal.
